# Supplementary material for: A lysozyme with altered substrate specificity facilitates prey cell exit by the periplasmic predator Bdellovibrio bacteriovorus
Source: Nat Commun. 2020 Sep 23;11:4817. doi: 10.1038/s41467-020-18139-8 (PMC7511926; doi:10.1038/s41467-020-18139-8)
Supplement: Supplementary file 1 — Supplementary Information [file 41467_2020_18139_MOESM1_ESM.pdf]

Supplementary Information

A Lysozyme with Altered Substrate Specificity Facilitates Prey Cell Exit by the Periplasmic Predator *Bdellovibrio bacteriovorus*

Harding *et al.*

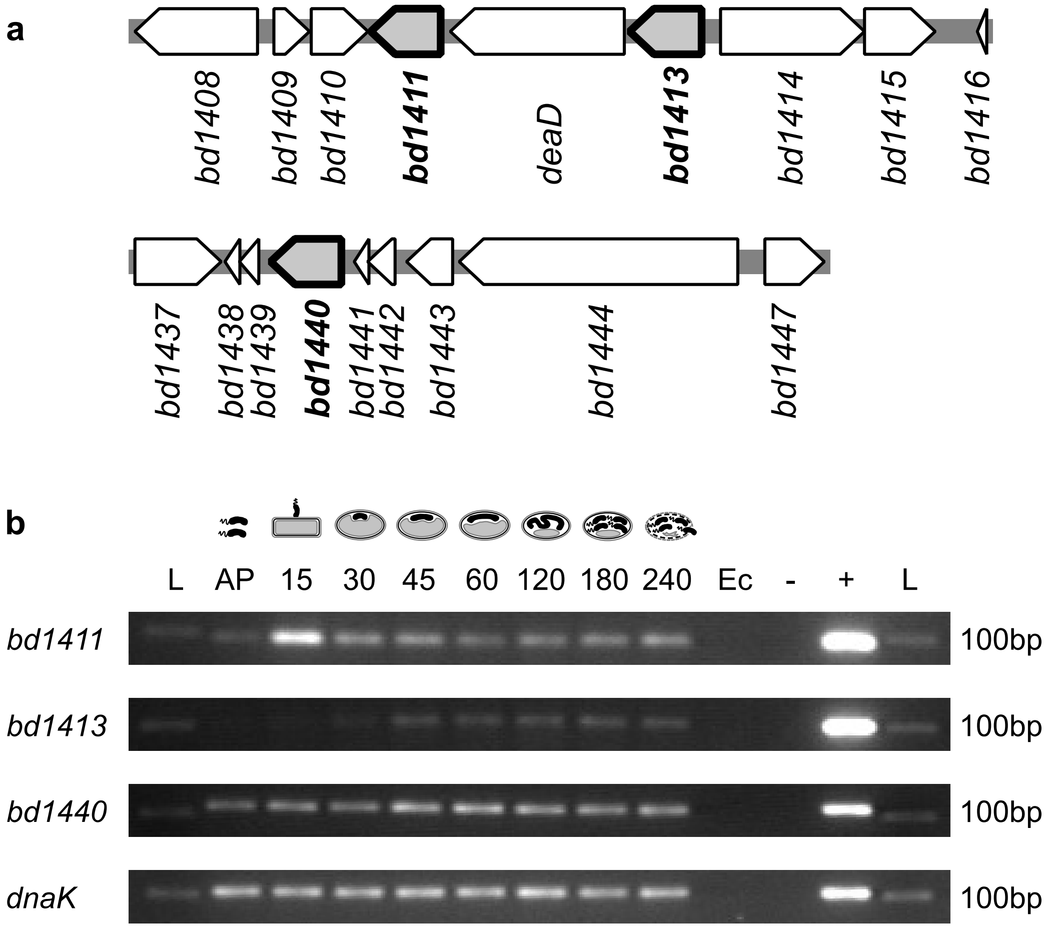

Supplementary Fig. 1 Differential expression of lysozyme homologue encoding genes *bd1411*, *bd1413* and *bd1440* during host-dependent lifecycle of *B. bacteriovorus* HD100. **a** Schematics of genomic context derived from xBASE[1] of cryptic lysozyme homologue genes *bd1411*, *bd1413* and *bd1440* including surrounding genes predicted to encode an outer membrane protein (*bd1408*), a Ni-Fe hydrogenase-I cytochrome b subunit (*bd1410*), an ATP-dependent RNA helicase (*deaD*), an ATP-binding protein in ABC transporter (*bd1414*), lysophospholipase (*bd1415*), a serine protease (*bd1444*) and hypothetical proteins (*bd1409*, *bd1416*, *bd1437*, *bd1438*, *bd1439*, *bd1441*, *bd1442*, *bd1443*, *bd1447*). **b** Transcriptional analysis by reverse transcriptase PCR showing the expression of other cryptic lysozymes *bd1411*, *bd1413* (*dslB)* and *bd1440* (*dslC)* over the host-dependent lifecycle of *B. bacteriovorus* HD100 as depicted in drawings above the agarose gel. AP: attack phase HD100 cells, 15-240: timepoint in minutes after invasion, Ec: *E. coli* S17-1 prey cells, -: negative control with H_2O, +: positive control with genomic DNA of *B. bacteriovorous* HD100, L: 100 bp DNA ladder. This figure shows one of two biological repeats with similar results. Source data for Supplementary Fig. 1b are provided in the Source Data file.

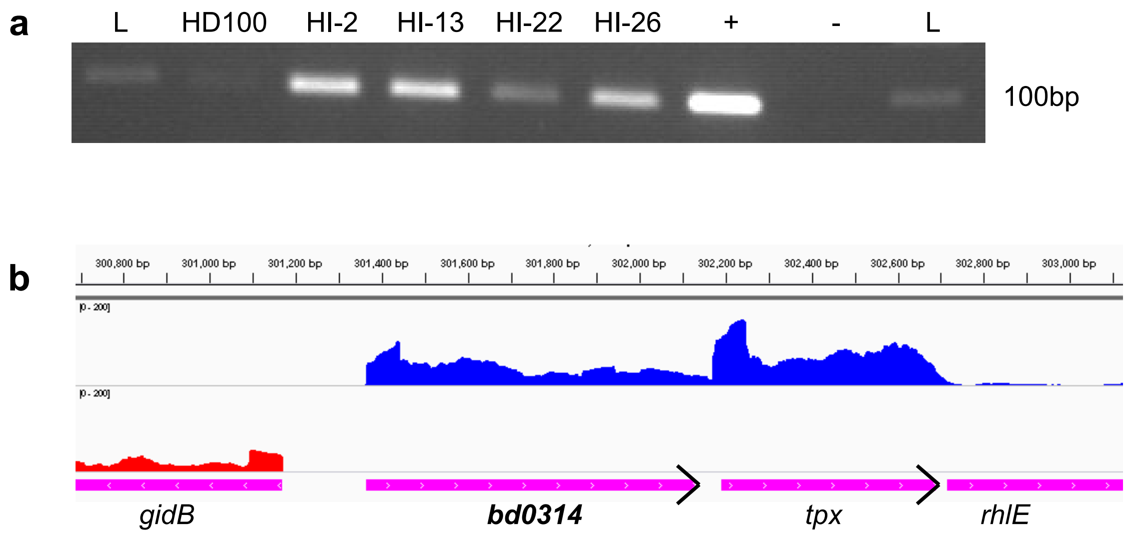

Supplementary Fig. 2 Gene *bd0314* is not transcribed in an operon with adjacent *tpx*. **a** Transcriptional analysis by Reverse Transcriptase PCR with primers binding within *bd0314* showing the general expression of *bd0314* in attack phase (outside prey) cells of *B. bacteriovorus* HD100 (AP) and different host-independently (HI) grown *B. bacteriovorus* strains (in which uncoordinated bdelloplast like growth, outside prey on axenic media is achieved due to different HI-causing point mutations) (HI-2, HI-13, HI-22, HI-26). +: positive control with genomic DNA of *B. bacteriovorous* HD100, -: negative control with H_2O, L: 100 bp DNA ladder (NEB). Expression of *bd0314* in all HI strains grown is seen in growing HI cells (OD_{600} of 0.6). Variability of expression levels between different HI strains is typical[2]. Source data for Supplementary Fig. 2a is provided in Source Data file. The four different HI strains shown here were tested with a similar result. **b** Transcriptional profile of *bd0314*. RNA-seq reads of *B. bacteriovorus* HID13 (HI strain derived from HD100, SRA accession PRJNA505601)[2,3,4] are mapped onto the genome sequence of *B. bacteriovorus* HD100[5] and represented in a blue bar chart. An initial steep rise of transcripts at the beginning of *dslA* and *tpx* suggest that the two genes are independently transcribed.

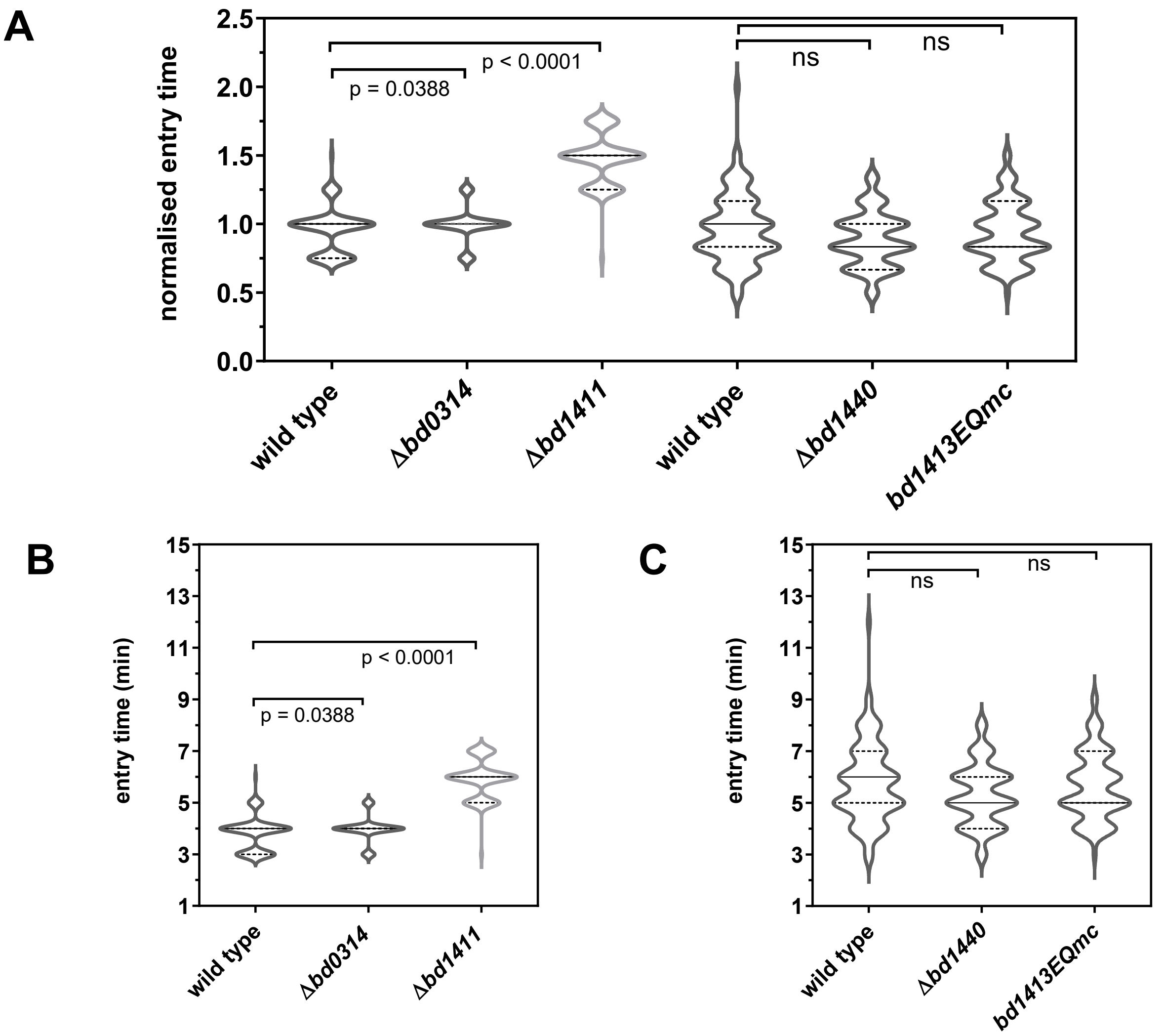

Supplementary Fig. 3 Δ*bd1411* shows a delayed entry phenotype. Violin plots of entry times of different *B. bacteriovorus* lysozyme mutant strains viewed by time-lapse microscopy on Nikon E600 microscope. Strains Δ*bd0314*, Δ*bd1411*, Δ*bd1440*, and *bd1413EQmc* were compared to *B. bacteriovorus* HD100 wild type upon invading *E. coli* S17-1 prey. **A** Normalised and (**B** and **C**) non-normalised entry times are shown. Experiments were performed in two batches (**B** and **C**) at ambient lab temperatures. Data were normalised for comparison. Normalisation in **A** was performed by division of each raw data point in **B** or **C** with the respective wild-type median value of the experimental batches (see accompanying raw data sheet). Entry videos were recorded with a 1 min frame rate causing discrete, (minute by minute) entry times. Data originate from at least three independent biological replicates. Within violin plots median values are a continuous horizontal line, while quartile lines are dashed. The two-tailed p-values are derived from the Mann-Whitney test (ns = non-significant, exact p-values are given directly in the figure). For source data of all 90 data points per violin plot and detailed histogram data of raw (non-normalised) data for violin plots **B** and **C** please refer to the Source Data File.

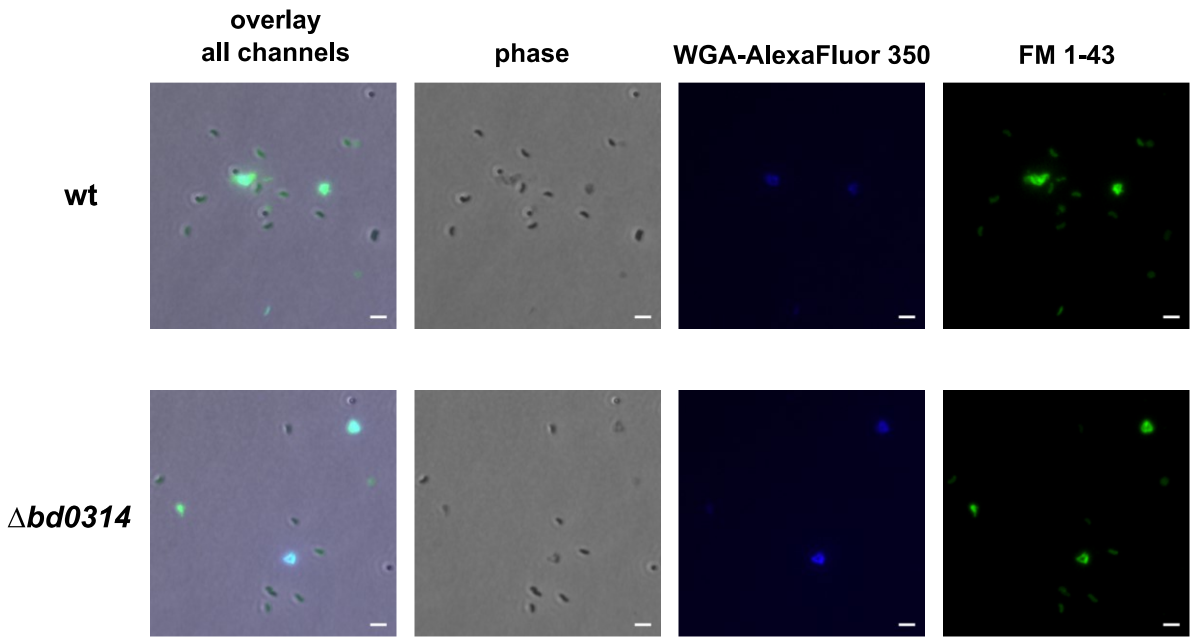

Supplementary Fig. 4 Example images of *B. bacteriovorus* HD100 wt and Δ*bd0314* at 5 hours post infection showing stained prey remnants. Samples were stained with Wheat Germ Agglutinin (WGA)-Alexa Fluor 350 conjugate (detection of prey peptidoglycan in DAPI channel) and FM 1-43 (detection of lipids in GFP channel). The brightness and contrast in the fluorescent channels was adjusted to increase prey remnant visibility. Scale bar is 2 μm. Four biological repeats were performed and showed similar results (Supplementary Figure 5).

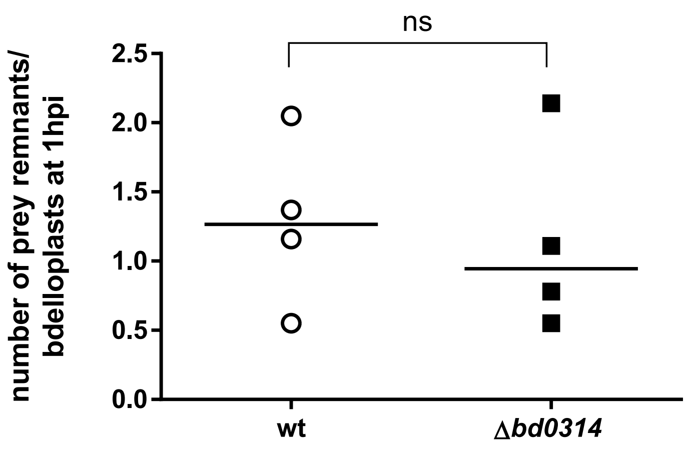

Supplementary Fig. 5 No obvious difference in number of prey remnants after *B. bacteriovorus* HD100 wild-type or Δ*bd0314* predation Prey remnants were evaluated after a semi-synchronous predatory life cycle at 5 hours post infection of *E. coli* K12 MG1655 prey with *B. bacteriovorus* HD100 wild type (empty circle) or Δ*bd0314* (filled square). (This 5 hr timepoint allowed the delayed prey exit of the Δ*bd0314* mutant to have been completed and debris compared to that of HD100 which exited around 4 hr). Peptidoglycan prey left-overs were stained with WGA-Alexa Fluor 350 conjugate for quantification. The ratio of WGA – Alexa Fluor 350 conjugate-stained prey remnants versus calibration polystyrene beads at 5 hours post infection was calculated and divided by the ratio of bdelloplasts (rounded prey cells infected with *Bdellovibrio*) to calibration polystyrene beads at 1 hour post-infection. Detailed raw data numbers are given in the Source Data file. Images were evaluated with Fiji[6,7] and MicrobeJ[8]. Four biological repeats are shown as points for each experiment. Statistical significance was tested with Mann-Whitney (two-tailed, ns = non-significant, lines represent median values).

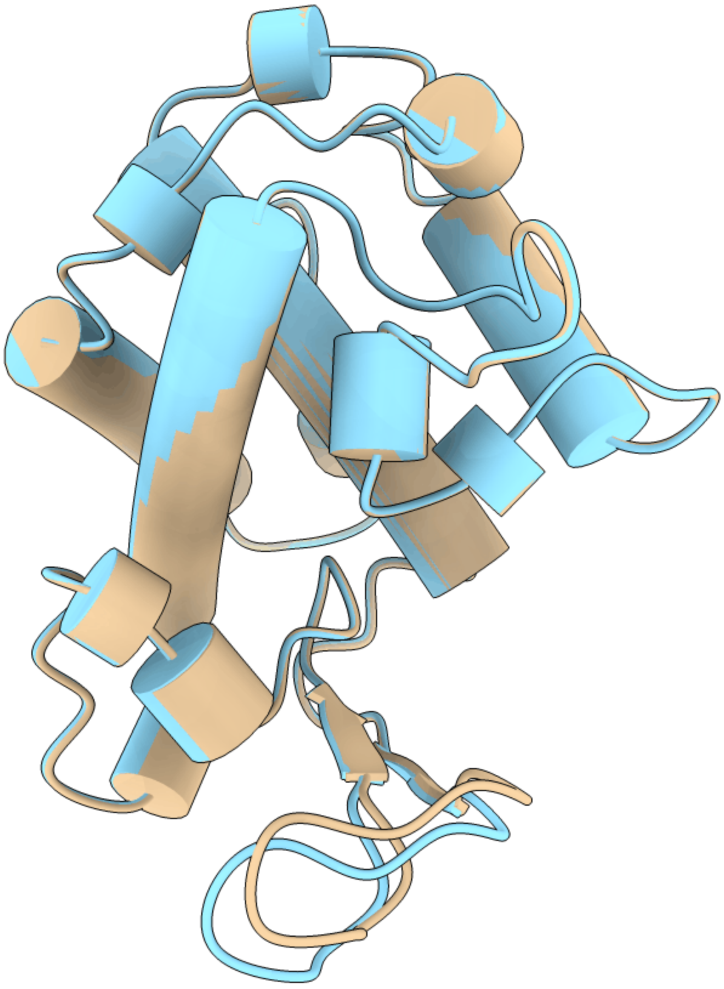

Supplementary Fig. 6 Comparison of representative wild-type DslA structure (tan colour) with E154Q variant (blue). The structures are largely identical, but there is a small but significant shift in the position/flexation of the β-hairpin that forms the lower region of the active site cleft as in Bd1413E151Qmcherry.

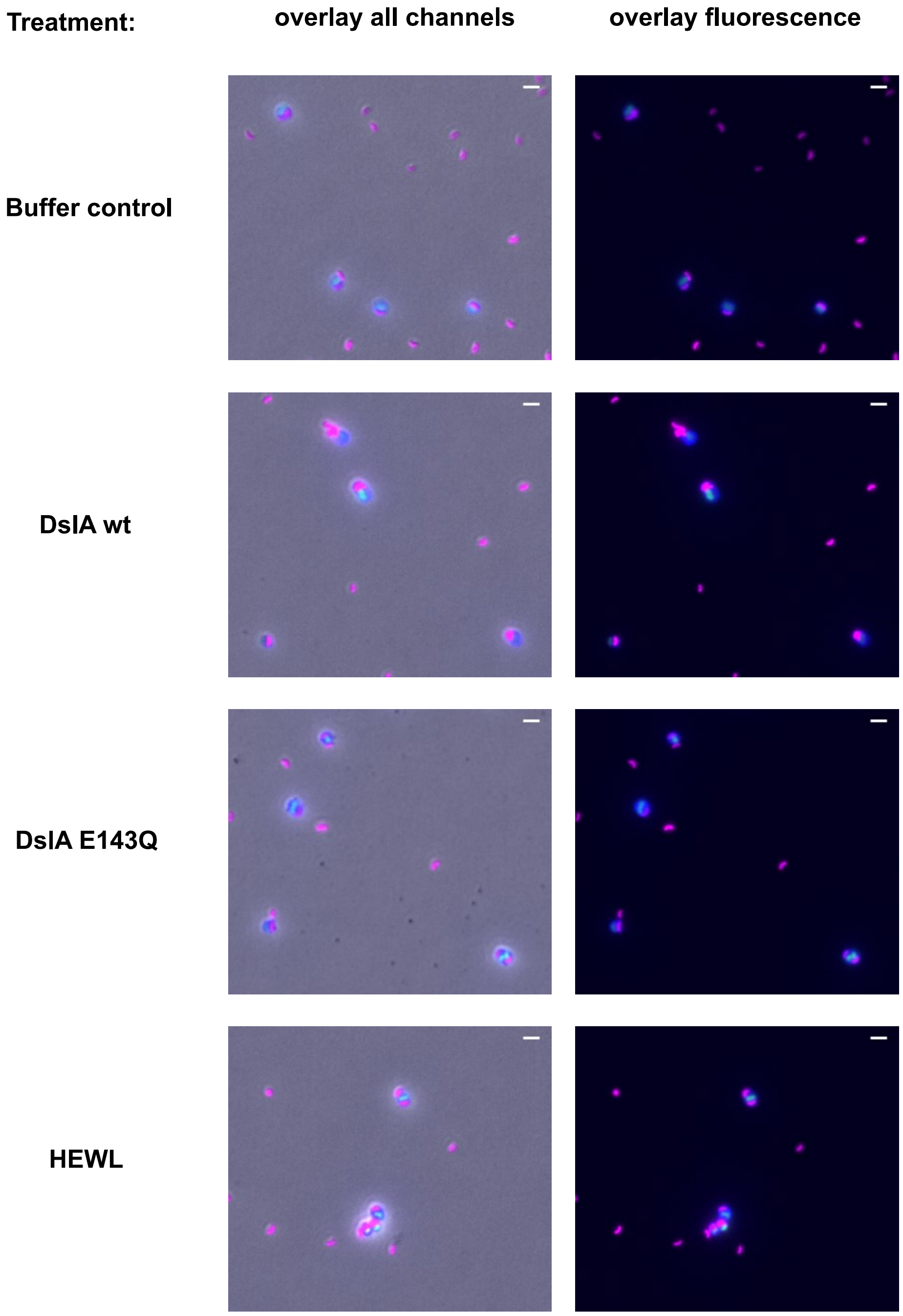

Supplementary Fig. 7 Example images of 1-hr bdelloplast release experiment by exogenous DslA or controls one hour after treatment. Images show all merged channels and the merge of all fluorescent channels including DAPI channel (for detection of HADA[9] integrated in peptidoglycan of prey *E. coli* S17-1 pAKF220), GFP channel (detection of mNeon in cytoplasm of prey *E. coli* S17-1 pAKF220) and mCherry (false coloured in magenta, detection of mCherry in cytoplasm of *B. bacteriovorus* HD100 *bd0064:mcherry*). The brightness and contrast in the fluorescent channels was adjusted to increase visibility. Dependent on treatment condition 3 to 5 biological repeats were performed with similar results (see Fig. 5c). Scale bar is 2 µm.

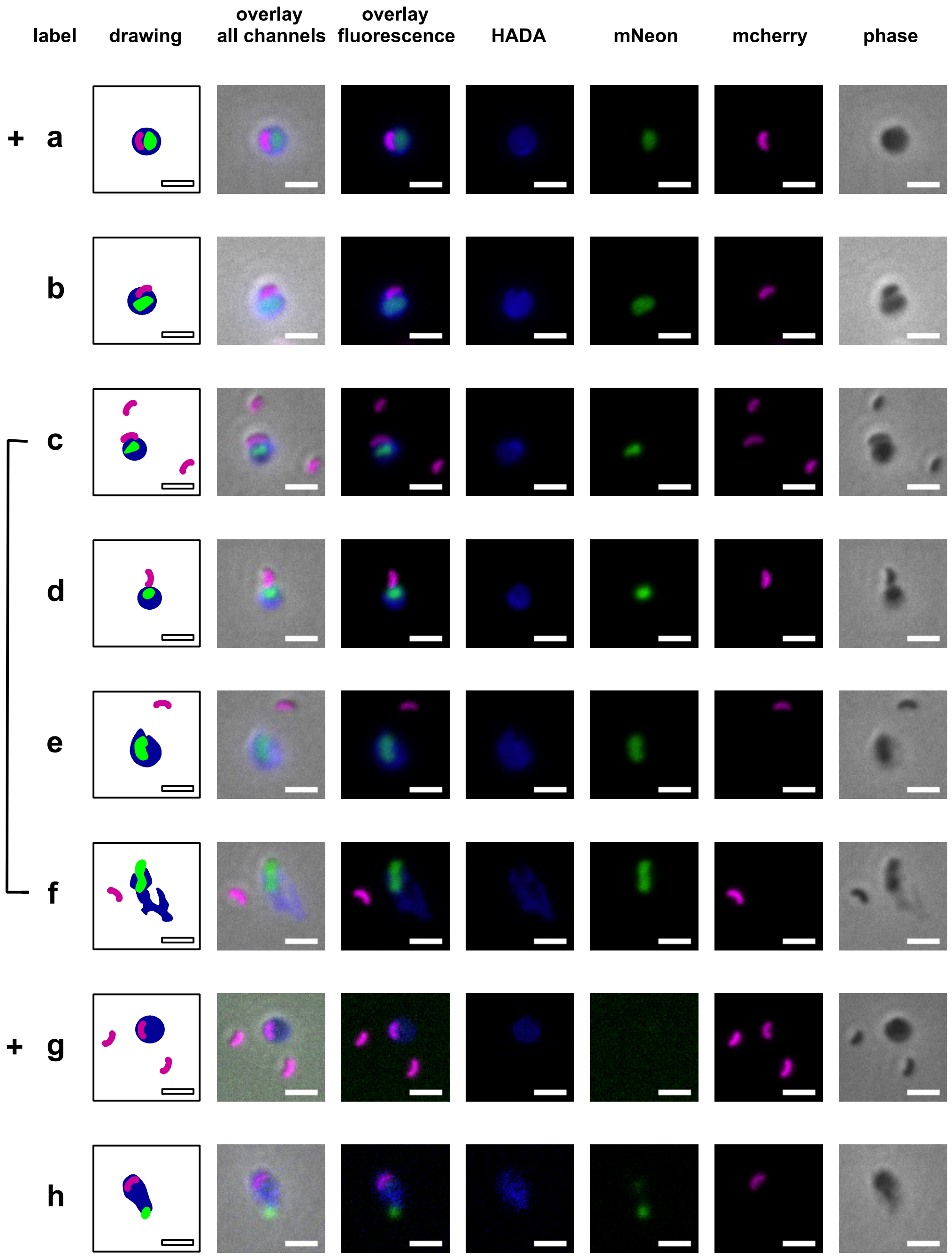

Supplementary Fig. 8 Categorisation of the different types of outcomes upon treatment of early bdelloplasts. A scheme of the experiment in which early bdelloplasts were treated by external addition of DslA is drawn in Fig. 5a. HADA[9] was used to pre-label peptidoglycan of prey *E. coli* S17-1 pAKF220 (expressing mNeonGreen in the cytoplasm). Predator *B. bacteriovorus* HD100 *bd0064:mcherry* exhibiting a fluorescent cytoplasm was mixed with the prey to form a bdelloplast. The 1-h bdelloplasts were treated for 10 minutes with 10 mM EDTA to permeabilise and then incubated with 10 μM DslA wt or a control (DslA E143Q, HEWL or Ca/HEPES buffer only) for one hour. Dependent on treatment condition 3 to 5 biological repeats were performed with similar results (see Fig. 5c). The treated bdelloplasts on

the microscope images were evaluated manually and sorted into categories with labels a-h. Brightness and contrast was adjusted individually if required to view and categorize each bdelloplast. Microscope images above show one typical example per category in all merged channels, merged fluorescent channels, the channels to detect HADA (peptidoglycan of prey *E. coli* S17-1 pAKF220 in DAPI channel, blue), mNeon (cytoplasm of prey *E. coli* S17-1 pAKF220 in GFP channel, green) and mCherry (cytoplasm of *B. bacteriovorus* HD100 *bd0064:mcherry* , mCherry, magenta), as well as phase contrast. The brightness and contrast of the images shown was adjusted to increase visibility. The same images of categories a, b and e are shown as examples again in Fig. 5. Scale bars are 2 µm. These categories were merged into the following four superordinate categories shown in Fig. 5 and Supplementary Fig. 9 and listed in the Source Data file: *Bdellovibrio* inside (category a & g, labelled with +) or outside (category c, d, e & f, labelled with bracket) the bdelloplast, *Bdellovibrio* partially inside and outside the bdelloplast (*Bdellovibrio* intermediate, category b) and *E. coli* cytoplasm is partially inside and partially outside the bdelloplast (*E. coli* cytoplasm intermediate, category h). Bdellovibrios attached to the outside of bdelloplasts without bdellovibrios in them as in category c and d could be freshly released bdellovibrios or new arrivals. For all experiments only single bdelloplasts (or their remnants, clearly not rod shaped) were evaluated. Upon treatment of early bdelloplasts with HEWL the bdelloplasts had a tendency to aggregate (Supplementary Fig. 7). In rare cases some sort of ‘shearing’ of the PG (detected by the fluorescent D-amino acid HADA[9]) could be detected on bdelloplasts after external addition of DslA and incubation for one hour under agitation (Supplementary Fig. 10).

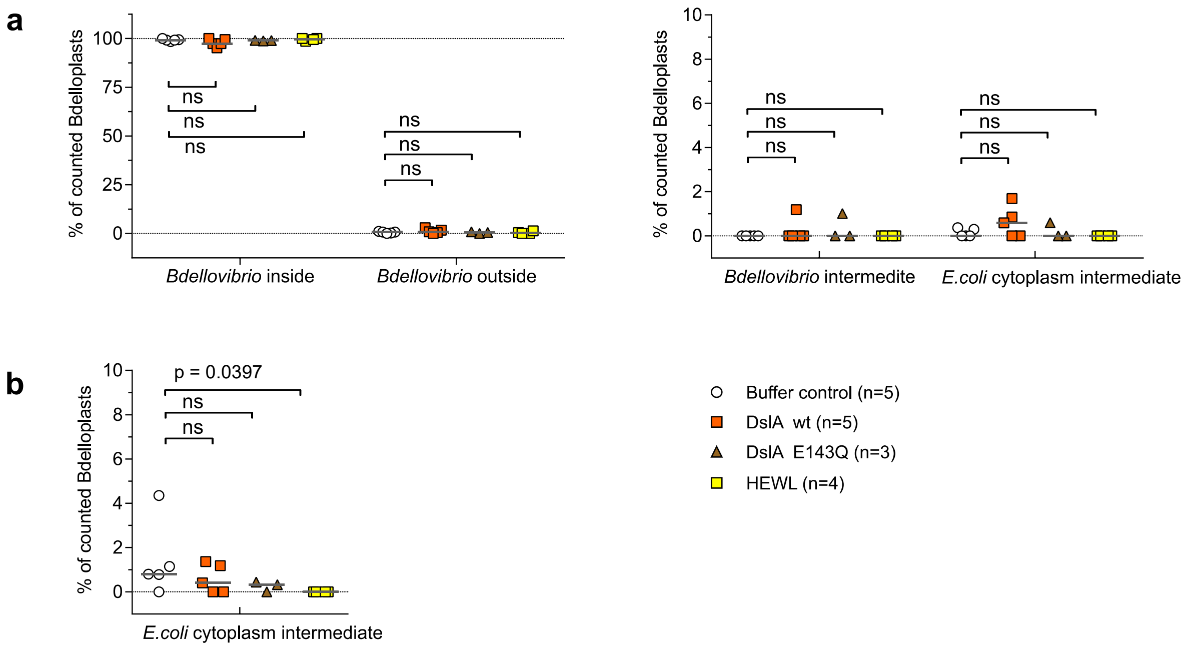

Supplementary Fig. 9 No differences of *B. bacterivorus bd0064:mcherry* location were seen at beginning of 1-hr bdelloplast release experiment. a Percentage of bdelloplasts categorized according to the position of the *Bdellovibrio* in respect to the bdelloplast before treatment as described (Fig. 5, Supplementary Fig. 8). **b** Percentage of bdelloplasts categorized according to the position of the *E. coli* cytoplasm in respect to the bdelloplast after treatment as described (Fig. 5, Supplementary Fig. 8). Categorization according to the position of *B. bacteriovorus* is shown in Figure 5. In very rare cases a leaking out of the prey *E. coli* cytoplasm from the bdelloplast was detected, and was not considered further as it was < 5% (one low significance value is due to a quadruple zero value in the control group). Individual symbols represent percentage of all categorized bdelloplasts per experiment. A minimum of 60 bdelloplasts (**a**) or 71 bdelloplasts (**b**) per treatment condition and biological repeat were evaluated. The number of biological repeats is indicated in the legend as n for the respective treatment. Horizontal lines represent median, statistical test was Mann-Whitney, ns = non-significant, p = 0.0397 (p-value is one-tailed). Source data are provided as a Source Data file.

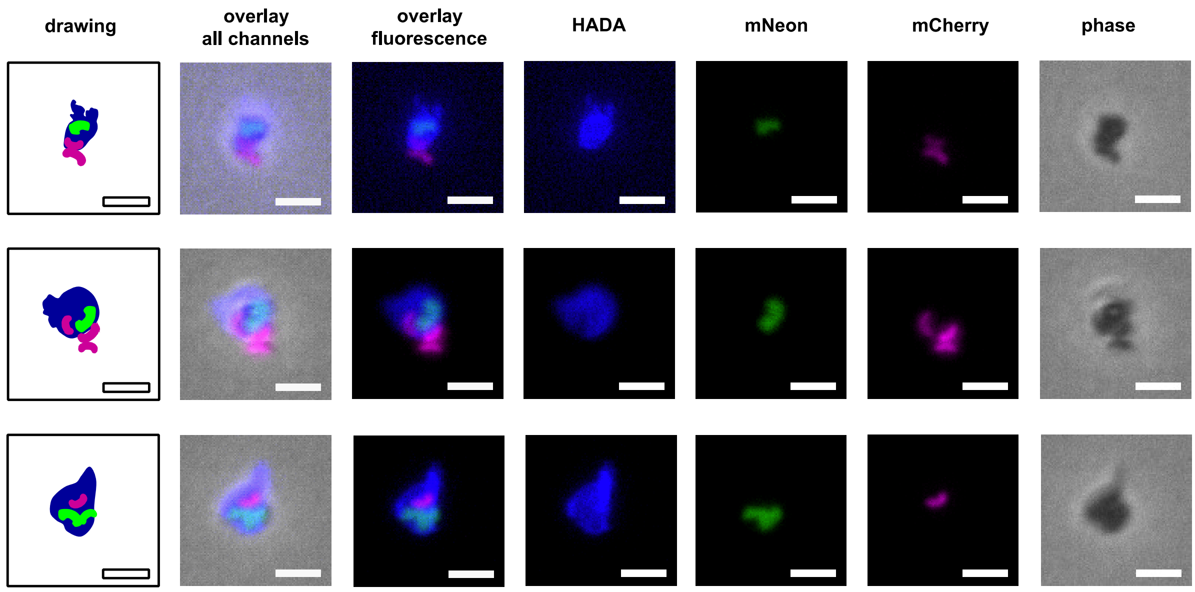

Supplementary Fig. 10 Example images where peptidoglycan is shearing off in samples where DslA wt was added externally. These examples were still categorised in ′a′ (Supplementary Fig. 8) based on the location of *B. bacteriovorus* HD100 *bd0064:mcherry* and the cytoplasm of *E. coli* S17-1 pAKF220 prey. Microscopic images show the overall overlay, the overlay of all fluorescent channels, the channels to detect HADA (peptidoglycan of prey *E. coli* S17-1 pAKF220 in DAPI channel), mNeon (cytoplasm of prey *E. coli* S17-1 pAKF220 in GFP channel) and mCherry (*B. bacteriovorus* HD100 *bd0064:mcherry*, mCherry false coloured in magenta), as well as phase contrast. The brightness and contrast in the fluorescent channels was adjusted to increase visibility. These images show some rare cases as pointed out in notes of Supplementary Fig. 8. Scale bars are 2 µm.

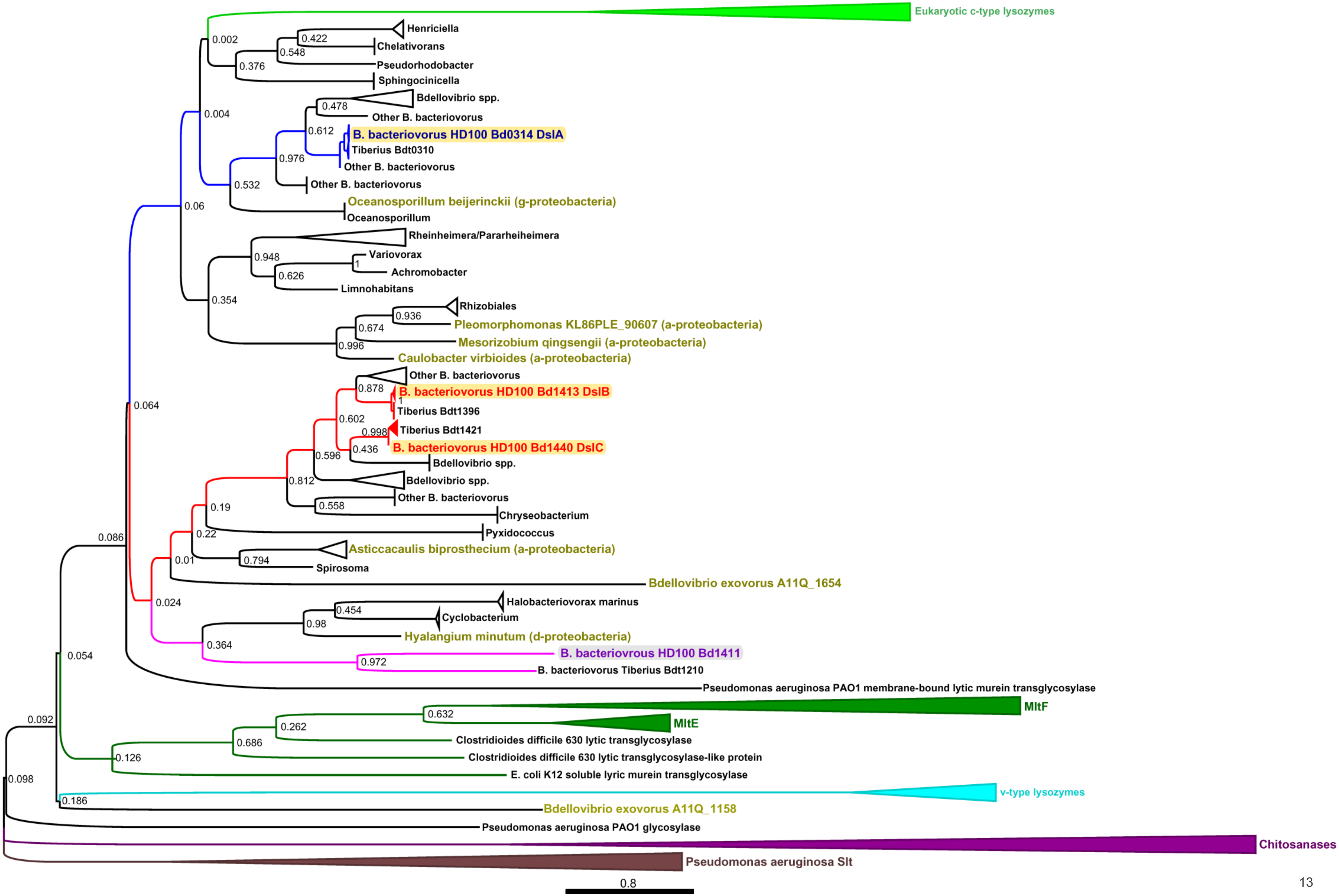
Eukaryotic c-type lysozymes
Henriciella
Chelativorans
Pseudorhodobacter
Sphingocinicella
Bdellovibrio spp.
Other B. bacteriovorus
B. bacteriovorus HD100 Bd0314 DslA
Tiberius Bdt0310
Other B. bacteriovorus
Other B. bacteriovorus
Oceanosporillum beijerinckii (g-proteobacteria)
Oceanosporillum
Rheinheimera/Pararheiheimera
Variovorax
Achromobacter
Limnohabitans
Rhizobiales
Pleomorphomonas KL86PLE_90607 (a-proteobacteria)
Mesorizobium qingsengii (a-proteobacteria)
Caulobacter virbioides (a-proteobacteria)
Other B. bacteriovorus
B. bacteriovorus HD100 Bd1413 DslB
Tiberius Bdt1396
Tiberius Bdt1421
B. bacteriovorus HD100 Bd1440 DslC
Bdellovibrio spp.
Bdellovibrio spp.
Other B. bacteriovorus
Chryseobacterium
Pyxidococcus
Asticcacaulis biprosthecium (a-proteobacteria)
Spirosoma
Bdellovibrio exovorus A11Q_1654
Halobacteriovorax marinus
Cyclobacterium
Hyalangium minutum (d-proteobacteria)
B. bacteriovrous HD100 Bd1411
B. bacteriovorus Tiberius Bdt1210
Pseudomonas aeruginosa PAO1 membrane-bound lytic murein transglycosylase
MltF
MltE
Clostridioides difficile 630 lytic transglycosylase
Clostridioides difficile 630 lytic transglycosylase-like protein
E. coli K12 soluble lyric murein transglycosylase
v-type lysozymes
Bdellovibrio exovorus A11Q_1158
Pseudomonas aeruginosa PAO1 glycosylase
Chitosanases
Pseudomonas aeruginosa Slt
0.8

Supplementary Fig. 11 (shown on previous page) A phylogenetic tree using protein sequences, showing the relationship between DslA (blue text and branches), DslB and DslC (red text and branches) as well as several other groups of lysozymes and related enzymes. These include c-type lysozymes (light green), lytic transglycosylases MltE and MltF (dark green), v-type lysozymes (light blue), chitosanases (purple) and *Pseudomonas aeruginosa* Slt lytic transglycosylases (brown). Additional sequences were added; Bd1411 (pink), potential Dsl homologues from Gram-negative bacteria and *B. exovorus* putative lytic transglycosylases (olive green text, *B. exovorus* sequences are non-Dsl due to lack of YW motif and other conserved features). Phyla shown in black text were Dsl homologues identified using NCBI BLAST program suite[10]. The tree was produced using MEGA-X software, utilising the Maximum Likelihood method with 500 bootstraps[11] (shown as the number at the nodes). FigTree software was used for visualisation of the tree.

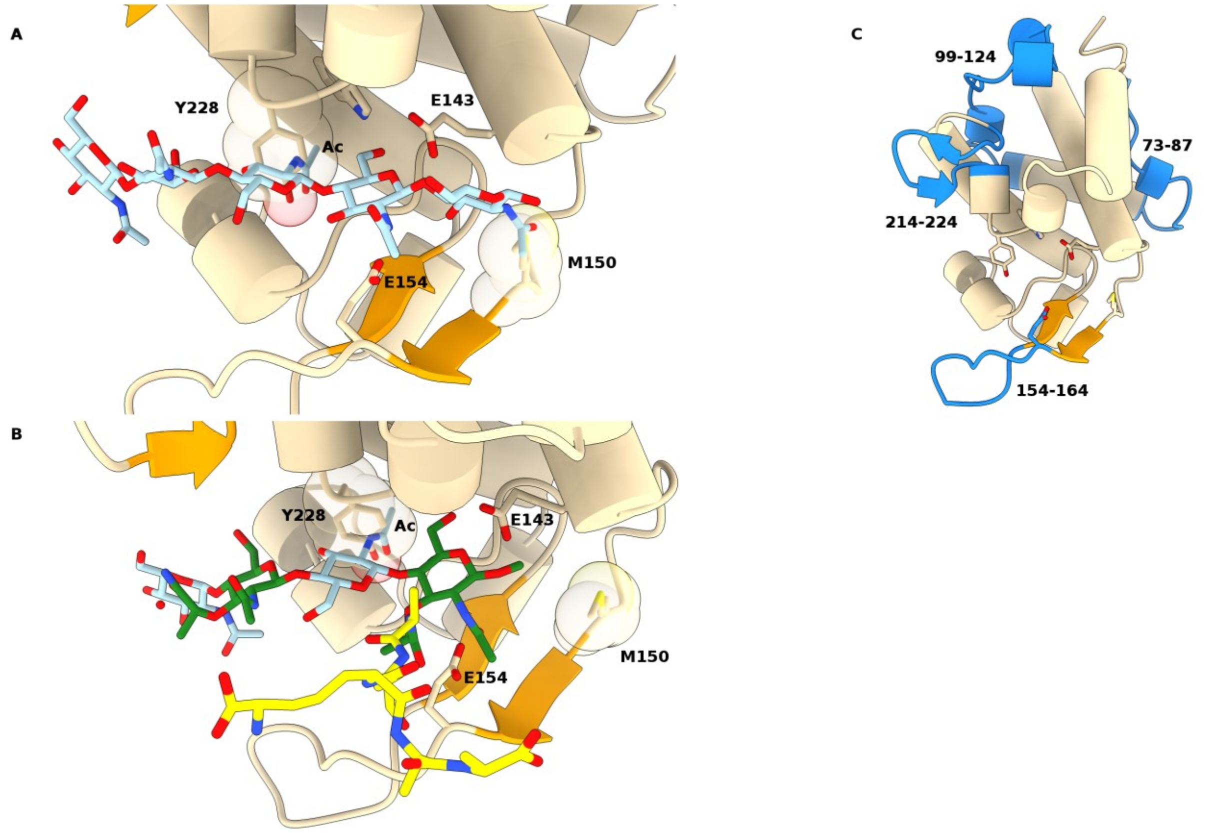

Supplementary Fig. 12 Additional substrate-binding models to confirm steric model for DslA substrate occlusion. Substrate-bound models were superimposed with the DslA structure in an identical manner to main text Figure 6 (via helix D and ES motif). Panel A shows the DslA structure and modelled substrate from PDB 4HJZ[12] (MltE, chitopentose); panel B shows DslA with a substrate from PDB 6FCS[13] (Slt, [NAG-NAMpentapeptide]$_2$), ligands in stick form, NAG blue, NAM green, peptide yellow. In both cases, Y228 of DslA occupies the same space as a NAG acetyl group. Panel C: regions of significant difference in fold (DslA comparison to lysozyme superfamily) are colored blue and the residue range numbered.

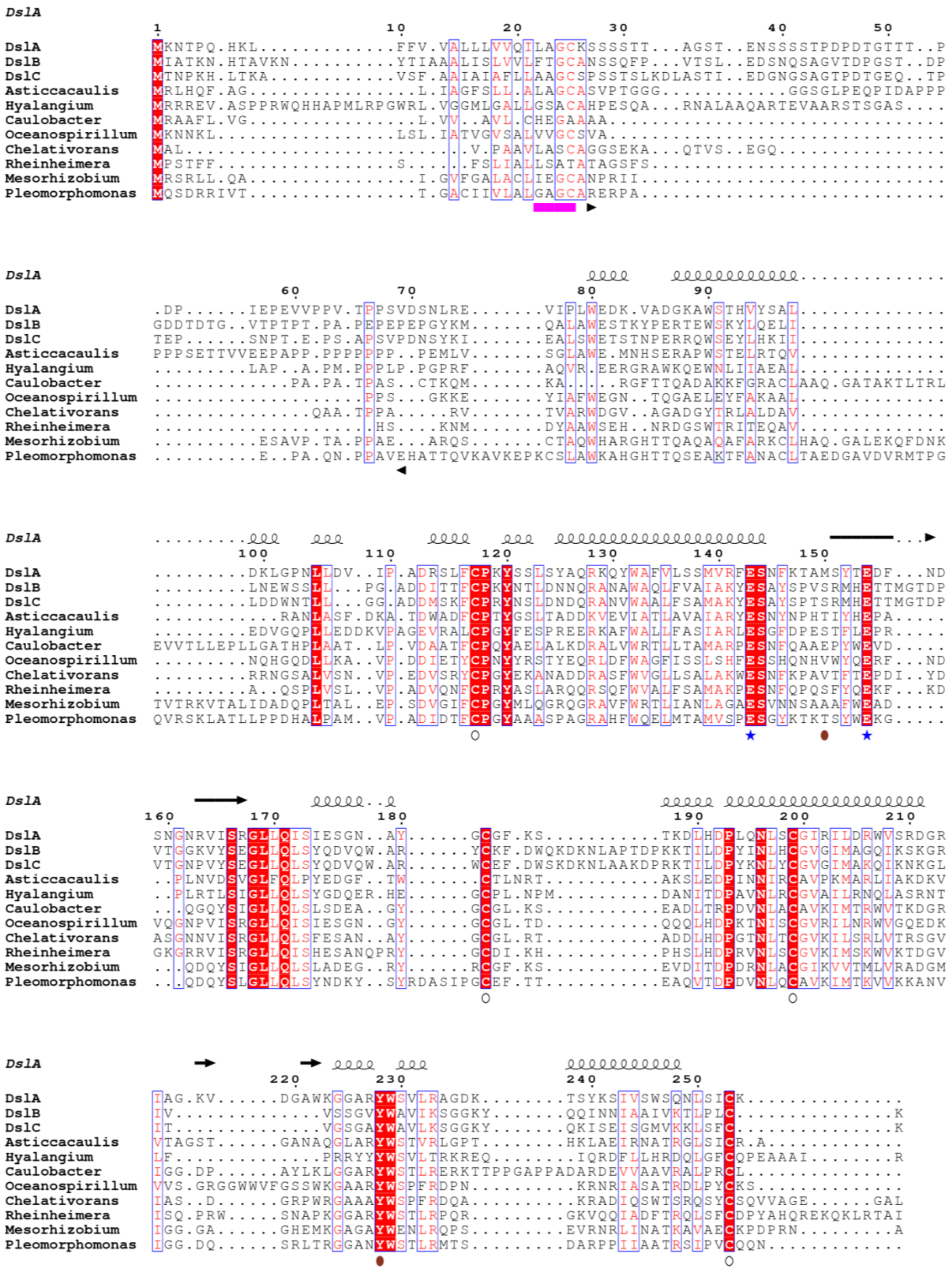

Supplementary Fig. 13 Sequence alignment of DslA and related enzymes. Sequences with significant homology to DslA were filtered to a representative set retaining conservation at both E154 and Y228. Key: lipobox, magenta bar; disordered region, ends delineated by black arrowheads; disulfides, open circles; catalytic residues, blue star; residues at 2' Ac in model, brown ellipse. Strictly conserved residues in red box, mostly conserved residues in transparent

box. Full sequence entries as follows: DslA (*Bdellovibrio bacteriovorus* HD100 *bd0314*, UniProt Q6MQY8); DslB (*B. bacteriovorus* HD100 *bd1413*, Q6MN49); DslC (*B. bacteriovorus* HD100 *bd1440*, Q6MN23); (*Asticcacaulis biprosthecium* C19 *ABI_41740*, F4QSN1); (*Hyalangium minutum*, NCBI WP_083968698); (*Caulobacter vibrioides* CB13b1a *CA606_11975*, A0A290MLW2); (*Oceanospirillum beijerinckii*, WP_051227967); (*Chelativorans sp.* BNC1, WP_041544544.1); (*Rheinheimera mesophila EIK76_01765*, A0A3P3QNM9); (*Mesorhizobium qingshengii*, WP_091585550); (*Pleomorphomonas sp. KL86PLE_90607*, A0A212LQ53). Figure prepared using ESPript[14].

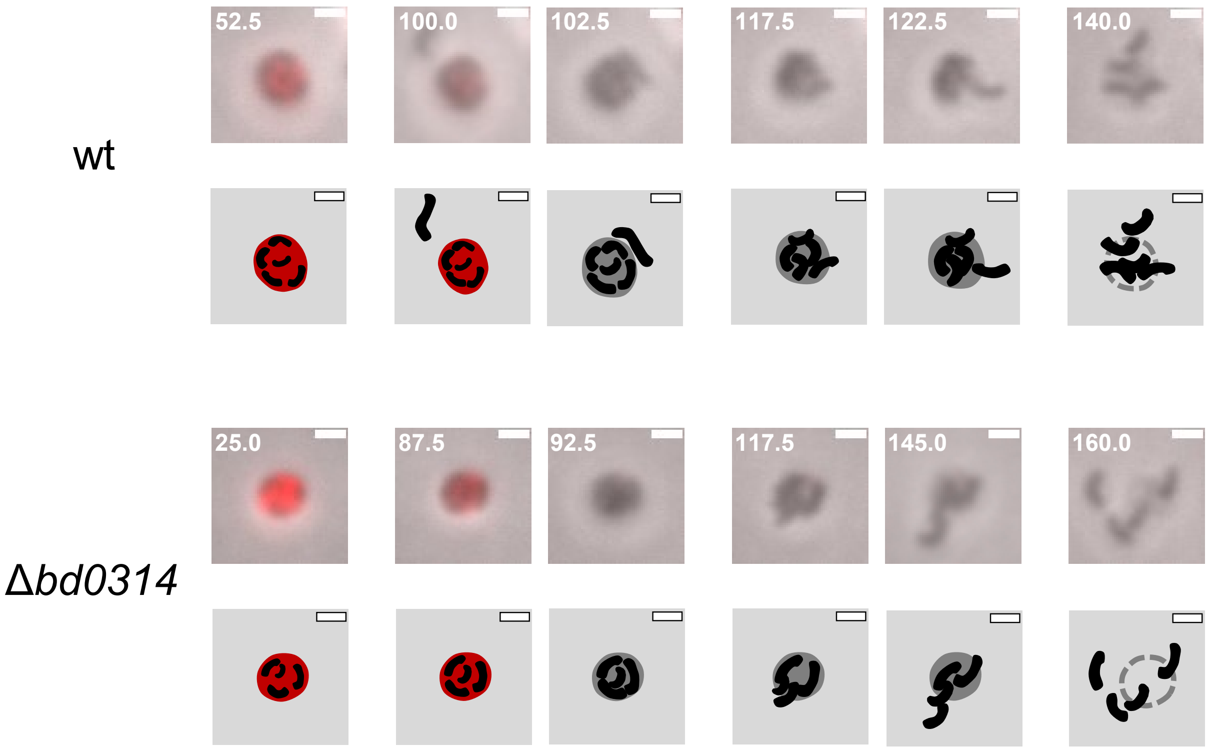

Supplementary Fig. 14 Epifluorescence time-lapse microscopy reveals no obvious difference in the exit mode between *B. bacteriovorus* HD100 wt and Δ*bd0314* apart from a delay (see Fig. 2). Bdelloplasts were placed on a 1% agarose slide 3 hours post infection of *E. coli* S17-1::pMAL-p2_mCherry (generating fluorescent prey periplasm and cytoplasm to determine backlit predator progeny septation[15]). Sample images show that prey fluorescence dissipates within 1-2 frames (< 5 min) as described in exit mode 1 of Lambert *et al.*[16]. No indication that fluorescent prey cytoplasm is left behind after exit of progeny cells of Δ*bd0314* was found in contrast to the double GlcNAc-deacetylase mutant[16]. Number at upper left of each image represents time in minutes after 3-hr bdelloplasts were placed on the agarose slide. Scale bars are 1 µm. A similar result with regards to exit mode was detected for all 45 evaluated bursting bdelloplasts of *B. bacteriovorus* HD100 wt or Δ*bd0314*.

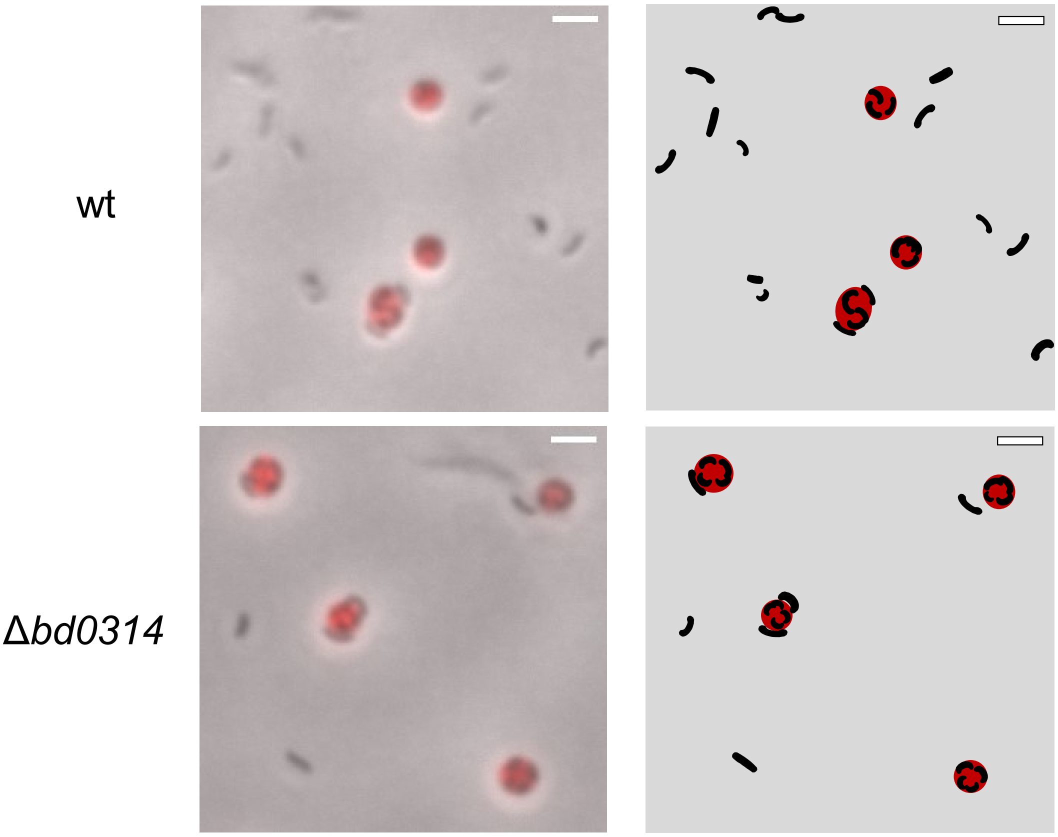

Supplementary Fig. 15 Single frames of epifluorescence time-lapse microscopy show no difference in predator progeny distribution between *B. bacteriovorus* HD100 wt and Δ*bd0314* in the bdelloplast. This is in contrast to the double GlcNAc-deacetylase mutant, where about half of the population assemble tighly between the prey cell wall and the outer membrane before exiting the bdelloplast[16]. Single frames presented here were taken around one hour after bdelloplasts, were placed on a 1% agarose slide for time-lapse microscopy 2hrs 45min after infection. Scale bars are 2 µm. A similar result with regards to exit mode/predator progeny distribution was detected for all 45 evaluated bursting bdelloplasts of *B. bacteriovorus* HD100 wt or Δ*bd0314*.

References for Supplementary Information:

1. Chaudhuri, R. R., Loman, N. J., Snyder, L. A. S., Bailey, C. M., Stekel, D. J. & Pallen, M. J. xBASE2: a comprehensive resource for comparative bacterial genomics. *Nucleic Acids Res.* **36**, D543-D546 (2008).

2. Capeness, M. J., et al. Activity of *Bdellovibrio hit* locus proteins, Bd0108 and Bd0109, links Type IVa pilus extrusion/retraction status to prey-independent growth signalling. *PLoS One* **8**, e79759 (2013).

3. Lambert, C., Ivanov, P. & Sockett, R. E. A transcriptional "Scream" early response of *E. coli* prey to predatory invasion by *Bdellovibrio*. *Curr. Microbiol.* **60**, 419-427 (2010).

4. Lowry, R. C., et al. Evolutionary diversification of the RomR protein of the invasive deltaproteobacterium, *Bdellovibrio bacteriovorus. Sci. Rep.* **9**, 5007 (2019).

5. Rendulic, S., et al. A predator unmasked: life cycle of *Bdellovibrio bacteriovorus* from a genomic perspective. *Science* **303**, 689-692 (2004).

6. Schindelin, J., et al. Fiji: an open-source platform for biological-image analysis. *Nat. Methods* **9**, 676-682 (2012).

7. Rueden, C. T., et al. ImageJ2: ImageJ for the next generation of scientific image data. *BMC Bioinformatics* **18**, 529 (2017).

8. Ducret, A., Quardokus, E. M. & Brun, Y. V. MicrobeJ, a tool for high throughput bacterial cell detection and quantitative analysis. *Nat. Microbiol.* **1**, 16077 (2016).

9. Kuru, E., et al. *In situ* probing of newly synthesized peptidoglycan in live bacteria with fluorescent D-amino acids. *Angew. Chem. Int. Ed. Engl.* **51**, 12519-12523 (2012).

10. Altschul, S. F., Gish, W., Miller, W., Myers, E. W. & Lipman, D. J. Basic Local Alignment Search Tool. *J. Mol. Biol.* **215**, 403-410 (1990).

11. Kumar, S., Stecher, G., Li, M., Knyaz, C. & Tamura, K. MEGA X: Molecular Evolutionary Genetics Analysis Across Computing Platforms. *Mol. Biol. Evol.* **35**, 1547-1549 (2018).

12. Fibriansah, G., Gliubich, F. I. & Thunnissen, A.-M. W. H., On the mechanism of peptidoglycan binding and cleavage by the endo-specific lytic transglycosylase MltE from *Escherichia coli*. *Biochemistry* **51**, 9164-9177 (2012).

13. Lee, M. et al. Exolytic and endolytic turnover of peptidoglycan by lytic transglycosylase Slt of *Pseudomonas aeruginosa*. *Proc. Natl. Acad. Sci. U. S. A.* **115**, 4393-4398 (2018).

14. Robert, X. & Gouet, P. Deciphering key features in protein structures with the new ENDscript server. *Nucleic Acids Res.* **42**, W320-324 (2014).

15. Fenton, A. K., Kanna, M., Woods, R. D., Aizawa, S. I. & Sockett, R. E. Shadowing the actions of a predator: backlit fluorescent microscopy reveals synchronous nonbinary septation of predatory *Bdellovibrio* inside prey and exit through discrete bdelloplast pores. *J. Bacteriol.* **192**, 6329-6335 (2010).

16. Lambert, C., et al. Interrupting peptidoglycan deacetylation during *Bdellovibrio* predator-prey interaction prevents ultimate destruction of prey wall, liberating bacterial-ghosts. *Sci. Rep.* **6**, 26010 (2016).
